# Prognostic value of pathogenic variants in Lafora Disease: systematic review and meta-analysis of patient-level data

**DOI:** 10.1186/s13023-023-02880-6

**Published:** 2023-09-02

**Authors:** Federica Pondrelli, Raffaella Minardi, Lorenzo Muccioli, Corrado Zenesini, Luca Vignatelli, Laura Licchetta, Barbara Mostacci, Paolo Tinuper, Craig W. Vander Kooi, Matthew S. Gentry, Francesca Bisulli

**Affiliations:** 1 Department of Biomedical and Neuromotor Sciences, University of Bologna, Bologna, Italy; 2 IRCCS Istituto delle Scienze Neurologiche di Bologna, Full Member of the European Reference Network for Rare and Complex Epilepsies (EpiCARE), Bologna, Italy; 3 Department of Biochemistry and Molecular Biology, University of Florida, Gainesville, USA

## Abstract

**Background:**

Lafora disease (LD) is a fatal form of progressive myoclonic epilepsy caused by biallelic pathogenic variants in *EPM2A* or *NHLRC1*. With a few exceptions, the influence of genetic factors on disease progression has yet to be confirmed. We present a systematic review and meta-analysis of the known pathogenic variants to identify genotype–phenotype correlations.

**Methods:**

We collected all reported cases with genetically-confirmed LD containing data on disease history. Pathogenic variants were classified into missense (MS) and protein-truncating (PT). Three genotype classes were defined according to the combination of the variants: MS/MS, MS/PT, and PT/PT. Time-to-event analysis was performed to evaluate survival and loss of autonomy.

**Results:**

250 cases described in 70 articles were included. The mutated gene was *NHLRC1* in 56% and *EPM2A* in 44% of cases. 114 pathogenic variants (67 *EPM2A*; 47 *NHLRC1*) were identified. The *NHLRC1* genotype PT/PT was associated with shorter survival [HR 2.88; 95% CI 1.23–6.78] and a trend of higher probability of loss of autonomy [HR 2.03, 95% CI 0.75–5.56] at the multivariable Cox regression analysis. The population carrying the homozygous p.Asp146Asn variant of *NHLRC1* genotype was confirmed to have a more favourable prognosis in terms of disease duration.

**Conclusions:**

This study demonstrates the existence of prognostic genetic factors in LD, namely the genotype defined according to the functional impact of the pathogenic variants. Although the reasons why *NHLRC1* genotype PT/PT is associated with a poorer prognosis have yet to be fully elucidated, it may be speculated that malin plays a pivotal role in LD pathogenesis.

**Supplementary Information:**

The online version contains supplementary material available at 10.1186/s13023-023-02880-6.

## Key points

- **What is already known on this topic**: Lafora disease (LD) is a fatal form of progressive myoclonic epilepsy caused by biallelic pathogenic variants in the *EPM2A* or *NHLRC1* genes. With a few exceptions, the influence of genetic factors on disease progression has yet to be confirmed.
- **What this study adds**: The study identified prognostic genetic factors in LD and demonstrated a correlation between certain genotypes and worse prognosis. Specifically, biallelic truncating variants in *NHLRC1* were associated with a higher probability of loss of autonomy and shorter survival. The study also confirmed that the homozygous p.Asp146Asn variant of *NHLRC1* has a more favourable prognosis.
- **How this study might affect research, practice, or policy**: The study sheds light on the potential genetic factors affecting the prognosis of LD, which could inform future research into treatments and therapies. This study’s findings should be taken into account when launching trials of disease-modifying therapies, to ensure that outcomes are correctly interpreted.

**Supplementary Information:**

The online version contains supplementary material available at 10.1186/s13023-023-02880-6.

## Introduction

Lafora Disease (LD, OMIM# 254780) is a rare autosomal recessive form of Progressive Myoclonus Epilepsy. It affects previously healthy children or adolescents, causing drug-resistant epilepsy, myoclonus, and psychomotor deterioration, leading to loss of autonomy and eventually death [1].

LD is caused by pathogenic variants in the *EPM2A* or *NHLRC1* (previously known as *EPM2B*) genes, causing the loss of function of the respective protein products involved in glycogen metabolism. *EPM2A* (6q24.3) consists of five exons encoding laforin, a 331-amino acid protein that contains a N-terminal carbohydrate-binding domain and a C-terminal dual-specificity phosphatase domain. *NHLRC1* (6p22.3) is an intronless gene that encodes a 395-amino acid protein named malin, an E3 ubiquitin ligase containing a zinc finger of the Ring type in its N-terminal region and six NHL-repeat domains in its C-terminal region.

Together, laforin and malin play an important role in regulating glycogen metabolism, thereby preventing the formation of polyglucosan aggregates [2]. When laforin or malin malfunctions, glycogen molecules become insoluble and precipitate, forming the so-called Lafora bodies (a type of polyglucosan aggregate) responsible for the dramatic clinical manifestations of LD. The physiologic functioning of these enzymes comprises both individual and interactive mechanisms and has not been fully elucidated thus far.

More than 150 different causative genetic variants in LD genes have been reported so far. These include point mutations, large deletions, and splicing mutations, giving rise to extreme allelic heterogeneity [3]. The majority of patients harbour compound heterozygous variants, as expected in rare recessive diseases when parents are non-consanguineous [4].

Recently, we published a prognostic meta-analysis of patient-level data [5] showing a median survival of 11 years and a median time to loss of autonomy of 6 years from disease onset. While also disclosing a great variability depending on age at onset and geographical origin (< 18 years and Asiatic origin were associated with shorter survival). On the other hand, the type of mutated gene and compound heterozygosity did not emerge as prognostic factors. This result is in contrast with previous observations describing a clear prognostic impact of specific genetic variants when harboured in the homozygous state [6–9].

However, considering loss-of-function gene variants, it is possible to recognize two main classes of mutated proteins: proteins carrying a missense mutation, which generally exhibit a normal molecular weight and, on the other side, lower molecular weight proteins (or the complete absence of the product) resulting from truncating variants (or gene deletion). Therefore, it could be hypothesized that the latter may provoke more detrimental effects on the physiological pathway and, as a result, a more severe phenotype [10].

Therefore, based on the updated body of evidence from the aforementioned systematic review [5], we performed an in-depth analysis of the mutations of the included cases and provided the results of the individual participants' data meta-analysis in order to further clarify the possible relationship between the type of genetic variants and the severity of the phenotype.

## Methods

The study was conducted in compliance with the reporting guidelines for prognostic systematic reviews and individual participant data meta-analysis. Individual participant data meta-analysis may be an appropriate methodological approach for summarizing data from case reports/case series, as suggested recently to support also the development of clinical practice guidelines recommendations in the field of rare neurological diseases. A protocol was registered in the PROSPERO database (CRD42020190877).

We performed a systematic literature search of the PubMed/MEDLINE and Embase databases using the combination of key terms already applied in our previous prognostic study on LD (Supplement) [5]. There was no restriction on the publication date. The last search was performed in March 2022. One reviewer (FP) selected relevant papers through title, abstract, and full-text screening. The reference lists of the identified articles were also reviewed to find additional references. We included in the analysis only genetically confirmed LD cases for which the specific pathogenic variant was reported. In addition, data on disease duration at the last follow-up was required for inclusion. Two independent reviewers (FP, LM) evaluated the selected reports and inserted the relevant clinical data in an ad hoc database. The opinion of a third reviewer (FB) was required to resolve any disagreement.

A molecular biologist (RM) reviewed the genetic data. The pathogenic variants in *EPM2A* and *NHLRC1* reported in the selected articles were divided into two groups: missense (MS) and protein-truncating (PT). The MS group comprised both missense pathogenic variants and small in-frame insertions/deletions, while the PT group included all the variants responsible for loss of function/haploinsufficiency as nonsense, frameshift, splice site variants, and partial or total gene deletions. As LD has an autosomal recessive pattern of inheritance, all the patients were then categorized according to their genotype in three different classes: MS/MS or PT/PT if they carried two variants of the MS or PT groups, respectively, or MS/PT if they were compound heterozygous for a variant included in the MS group and the other variant included in the PT group.

### Statistical analysis

For the descriptive analysis stratified by gene (*EPM2A* and *NHLRC1*), continuous variables are presented as mean ± standard deviation (SD), and categorical variables as absolute and relative frequency (%). The Kaplan–Meier estimate was used to calculate the cumulative time-dependent probability of death or loss of autonomy. The time of entry into the analysis was taken as the year of onset, while the time of the endpoint was the year of death or of loss of autonomy, or the year of the last follow-up information (truncated at 15 years of follow-up), whichever came first. Univariable and multivariable Cox regression models with mixed effects (clustered survival data) were employed to study the association between time to death or time to loss of autonomy and the three different variant classes (MS/MS vs. MS/PT vs. PT/PT) for the two genes separately. We included in the multivariable model only those variables that were significant in the univariable models.

Since several reports described a slower disease course in patients harbouring the homozygous p.Asp146Asn (D146N) variant in *NHLRC1* [6–9], we decided to verify the prognostic features of this population by performing a sub-group analysis.

Lastly, we performed a post-hoc analysis combining the two genetic groups using as categories the classes MS/MS and MS/PT of *NHLRC1*, PT/PT of *NHLRC1*, and all variant classes of *EPM2A*. We included geographic origin and age at onset as independent variables in the multivariable Cox regression models.

The analysis was performed using data at the single-patient level. The included studies were considered in the models as cluster variables. The results are presented in Kaplan–Meier curves with hazard ratios (HR) and 95% confidence intervals (95% CIs). The assumption of proportional hazard was assessed by Schoenfeld residuals (p > 0.05). Statistical analysis was performed with the Stata SE statistical package, version 14.2.

To facilitate the interpretation of data, we used the following terms:

- “Death” and “Loss of autonomy” in the tables showing the results of the univariable and multivariable analysis.
- “Survival” and “Retention of autonomy” in the figures.

## Results

### Description of the analysis population

**Fig. 1:**
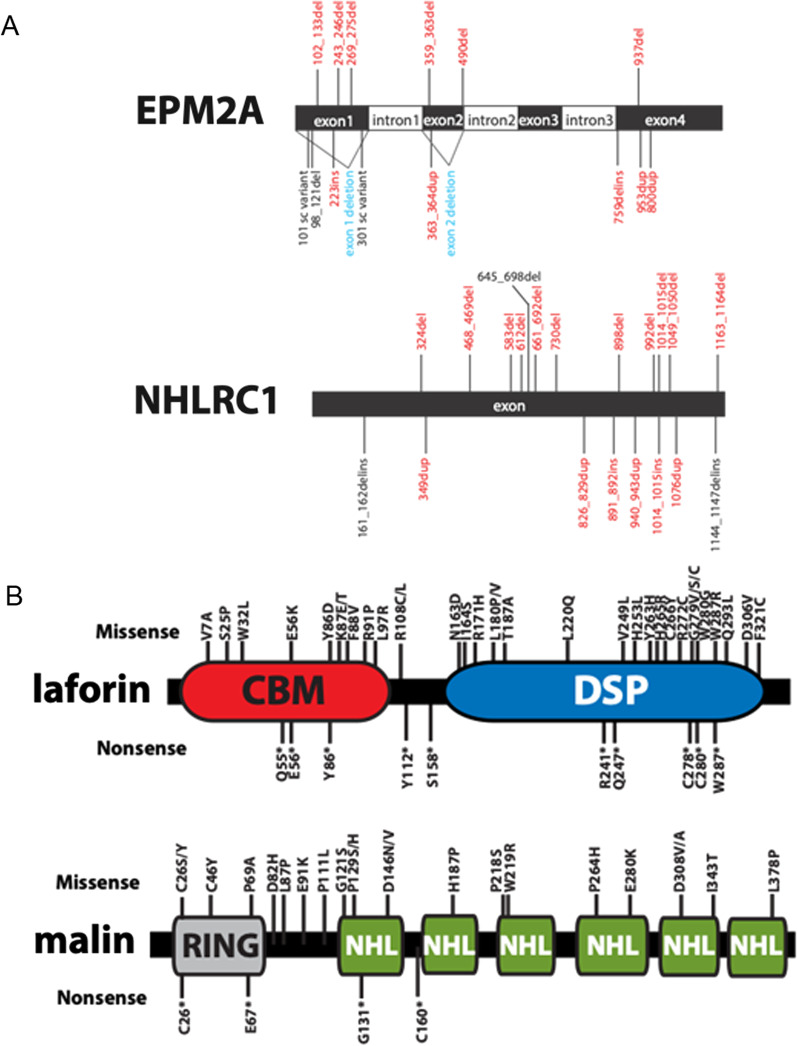
Schematic representation of the Lafora disease pathogenic variants in *EPM2A* and *NHLRC1* (**A**), laforin and malin (**B**). *EPM2A* gene contains five exons and *NHLRC1* consists of one exon. Laforin contains an amino-terminal carbohydrate binding module (CBM) and a carboxy-terminal dual specificity phosphatase domain (DSP). Malin contains a RING finger domain and six NHL repeats

We identified 70 publications reporting on 250 genetically confirmed cases that were eligible for inclusion in the final analysis. The selection process is depicted in a flow diagram (Additional file 1: Fig. S1) and all included references are reported in the Supplementary Text. The publication date of the included articles ranged from 2002 to 2022. Table 1 summarizes the clinical and genetic features of the included patients. Overall, we identified 67 different variants in *EPM2A* and 47 in *NHLRC1* (Additional file 2: Table S1 and S2; Fig. 1); 141 (56.4%) of the 250 patients carried pathogenic variants in *NHLRC1,* whereas 109* (*43.6%) had variants in *EPM2A*. Concerning genotypes, PT/PT prevailed for *EPM2A* variants (53.2% of the cases), while MS/MS was the most represented among *NHLRC1* variants (53.2% of the cases). The percentage of compound heterozygous MS/PT variation was around 28% for both genes.

**Table 1:** Clinical and genetic features of LD patients according to causative gene

Characteristics	*EPM2A*	*NHLRC1*
N	109/250 (43.6%)	141/250 (56.4%)
Sex, male	37/87 (42.5%)	44/102 (43.1%)
Sex, female	50/87 (57.5%)	58/102 (56.9%)
Geographic origin		
European	48/109 (44.0%)	93/141 (66.0%)
Asian	33/109 (30.3%)	36/141 (25.5%)
American	25/109 (22.9%)	9/141 (6.4%)
African	3/109 (2.8%)	3/141 (2.1%)
Family history		
Number of families/cases	87/109	121/141
Consanguinity	44/109 (40.4%)	45/141 (31.9%)
Age at disease onset		
Mean	12.7 ± 3.8 [4–28] in 109	14.1 ± 3.7 [5–30] in 141
Compound heterozygosity	30/109 (27.5%)	39/141 (27.7%)
Genotypes		
MS/MS	35/109 (32.1%)	75/141 (53.2%)
Compound Het	3/35 (8.6%)	14/75 (18.7%)
MS/PT	16/109 (14.7%)	16/141 (11.3%)
Compound Het	16/16 (100%)	16/16 (100%)
PT/PT	58/109 (53.2%)	50/141 (35.5%)
Compound Het	11/58 (19.0%)	9/50 (18.0%)
Loss of autonomy at last follow up	35/52 (67.3%)	48/64 (75.0%)
Mean age at onset	18.2 ± 4.7 [11–40] in 35	20.5 ± 6.8 [12–42] in 48
Mean time from disease onset	6.6 ± 4.4 [0.5–23] in 35	6.9 ± 5.6 [0.2–21] in 48
Deceased at last follow up	27/109 (24.8%)	42/141 (29.8%)
Mean age at death	22.4 ± 8.9 [14–59] in 27	21.1 ± 3.0 [16–35] in 42
Mean disease duration	9.4 ± 7.5 [3–40] in 27	7.5 ± 3.1 [2–23] in 42

n/N (%) or Mean (SD) [range], yr

*n/N* number of cases in which a certain characteristic is present out of the total number of cases which it was described, *SD* standard deviation

### Time to death and loss of autonomy in *EPM2A* variant-related cases

Twenty-seven of the 109 patients with an *EPM2A* variant (24.8%) were deceased as of their last follow-up (Table 1). The mean age at death was 22.4 years. Overall survival rates were 92% [95% CI 84–96] at 5 years, 59% [95% CI 44–72] at 10 years, and 49% [95% CI 32–64] at 15 years.

Information on autonomies in daily life activities was available for 52 patients; among them, 35 (67%) exhibited a loss of autonomy (Table 1). Out of the 35 patients for whom data on age at loss of autonomy and duration of the disease at that point were available, the mean age was found to be 18.2 years following a mean disease duration of 6.6 years (Table 1). The probability of retaining autonomy was 72% [95% CI 57–83] at 5 years, 17% [95% CI 6–32] at 10 years, and 4% [95% CI 0–18] at 15 years.

**Fig. 2:**
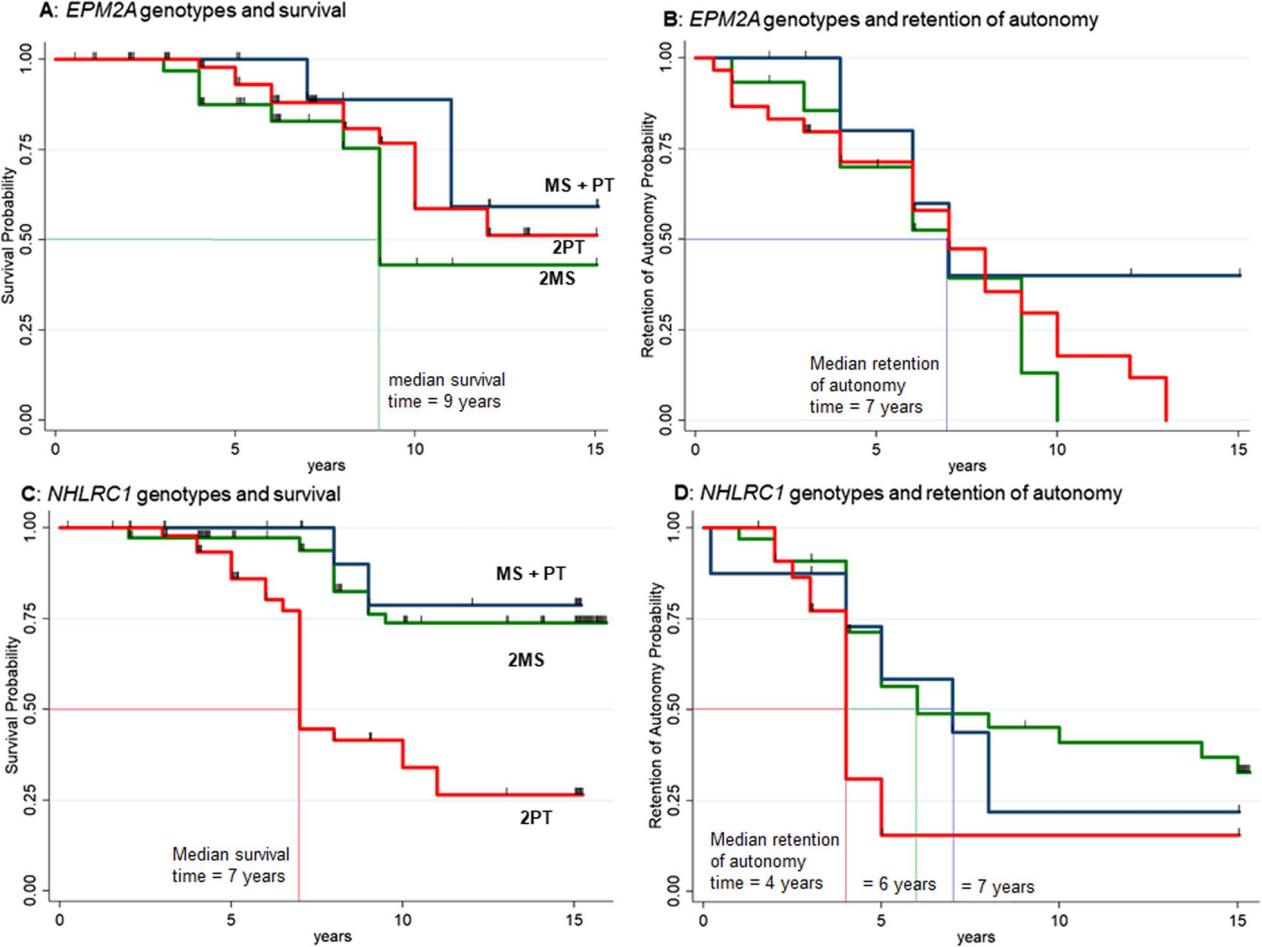
Association between genotypes and survival (*EPM2A*, **A**; *NHLRC1*, **B**) and retention of autonomy (*EPM2A*, **C**; *NHLRC1*, **D**) according to Kaplan–Meier analysis. Legend: MS, missense pathogenic variants and small in-frame insertions/deletions; PT, nonsense, frameshift, splice site variants, and partial or total gene deletions. MS + PT: genotype composed by one MS variant and one PT variant; 2MS: genotype composed by two MS variants; 2PT: genotype composed by two PT variants

Multivariable analysis showed no differences in death and loss of autonomy between the various genotypes. Asian and American origin were associated with higher probability of death compared to European origin, and Asian patients also showed a higher probability of loss of autonomy compared to European cases (Table 2). The association between *EPM2A* genotypes and death/loss of autonomy is presented in Fig. 2A, B.

**Table 2:** Association of variables with death and loss of autonomy in *EPM2A* and *NHLRC1-*related LD: multivariable analysis

Variables	Death (n = 109)		Loss of autonomy (n = 52)	
	HR (95% CI)	P value	HR (95% CI)	P value
*EPM2A*				
MS/MS	Reference	-	Reference	-
MS/PT	0.37 (0.06–2.03)	0.25	0.27 (0.07–1.09)	0.066
PT/PT	0.58 (0.21–1.62)	0.29	0.74 (0.34–1.64)	0.46
Age at onset 18 + versus < 18 years	0.65 (0.10–4.29)	0.66	4.16 (0.87–19)	0.073
*Geographic origin*				
Asian versus European	**7.60 (2.62**–**22.0)**	** < 0.001**	**4.83 (1.58**–**14.9)**	**0.006**
African versus European	–*	–	–*	–
American versus European	**3.91 (1.26**–**12.2)**	**0.018**	2.70 (0.90–8.11)	0.076

Variables	Death (n = 141)		Loss of autonomy (n = 64)	
	HR (95% CI)	P value	HR (95% CI)	P value
*NHLRC1*				
MS/MS	Reference	–	Reference	–
MS/PT	0.56 (0.12–2.68)	0.47	1.24 (0.46–3.32)	0.68
PT/PT	2.66 (1.10–6.39)	0.029	2.12 (1.04–4.31)	0.038
Age at onset 18 + versus < 18 years	0.20 (0.02–1.69)	0.14	0.41 (0.10–1.76)	0.23
*Geographic origin*				
Asian versus European	1.17 (0.31–4.37)	0.82	1.10 (0.15–7.82)	0.93
African versus European	3.78 (0.21–67.2)	0.37	9.23 (0.24–354)	0.23
American versus European	0.86 (0.07–10.5)	0.91	0.82 (0.12–5.36)	0.83

Bold text indicates statistical significance

*Estimation was not possible due to everyone surviving (n = 3)

### Time to death and loss of autonomy in *NHLRC1* variant-related cases

Forty-two of the 141 patients with an *NHLRC1* variant (29.8%) were deceased as of their last follow-up (Table 1) at a mean age of 21.1 years. Overall survival rates were 94% [95% CI 88–97] at 5 years, 61% [95% CI 51–70] at 10 years, and 58% [95% CI 47–68] at 15 years.

Information on autonomies in daily life activities was available for 64 patients; among them, 48 (75%) exhibited a loss of autonomy (Table 1). Out of the 48 patients for whom data on age at loss of autonomy and duration of the disease at that point were available, the mean age was found to be 20.5 years, following a mean disease duration of 6.6 years (Table 1). The probability of retaining autonomy was 43% [95% CI 30–56] at 5 years, 30% [95% CI 18–43] at 10 years, and 25% [95% CI 14–38] at 15 years.

Multivariable analysis revealed that the PT/PT genotype was associated with a higher probability of death [HR 2.66; 95% CI 1.10–6.39] and a higher probability of loss of autonomy [HR 2.12; 95% CI 1.04–4.31] (Table 2). Geographic origin did not affect survival or loss of autonomy in LD arising from *NHLRC1* variants. The association between *NHLRC1* genotypes and death and loss of autonomy is presented in Fig. 2C, D. The p.Asp146Asn variant in *NHLRC1* was present in the homozygous state in 18 subjects. The sub-group analysis showed that this variant is significantly (p < 0.001) associated with a higher probability of survival in comparison with the other *NHLRC1* genotypes, with a disease duration > 15 years in all the patients (Additional file 1: Fig. S2).

### Time to death and loss of autonomy in both *NHLRC1* and *EPM2A*-related cases combined

**Fig. 3:**
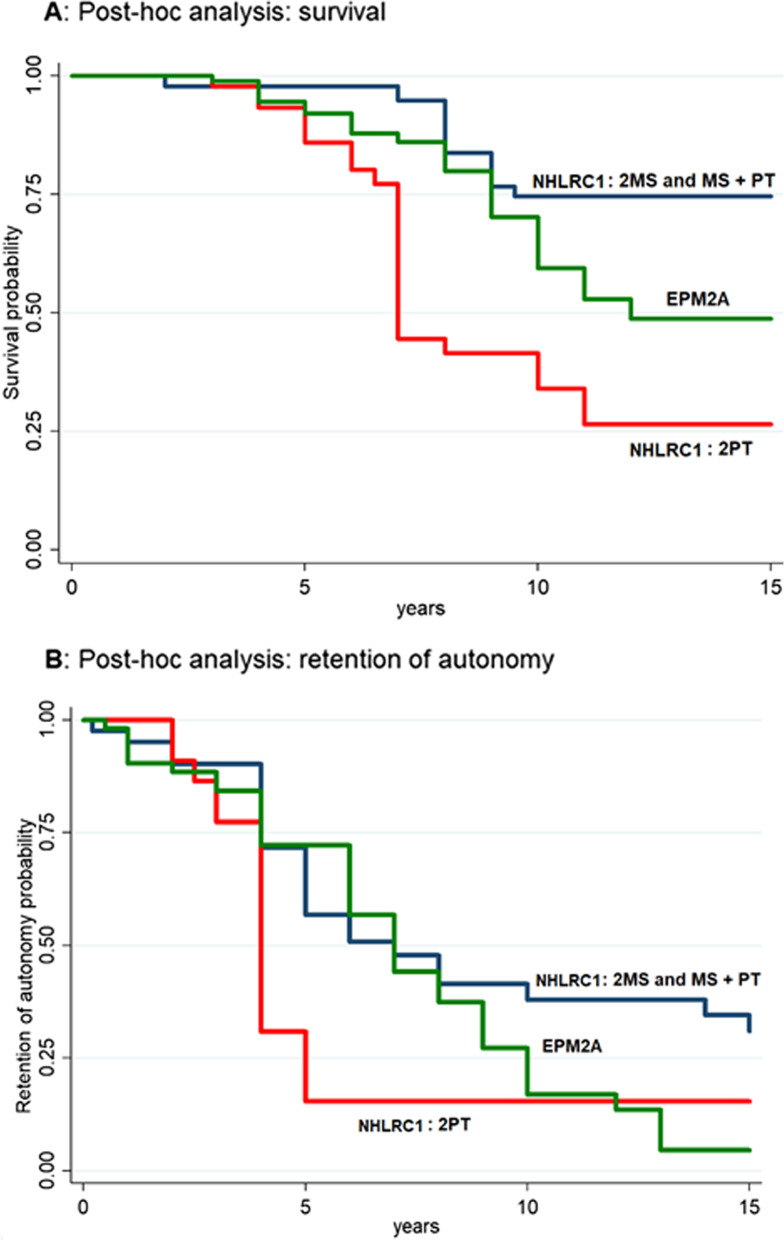
Post-hoc analysis comparing survival (**A**) and probability of retention of autonomy (**B**) between *EPM2A* genotypes taken together, *NHLRC1* genotype composed by two PT variants (2PT) and the other *NHLRC1* genotypes taken together (2MS and MS + PT). Legend: MS, missense pathogenic variants and small in-frame insertions/deletions; PT, nonsense, frameshift, splice site variants, and partial or total gene deletions. MS + PT: genotype composed by one MS variant and one PT variant; 2MS: genotype composed by two MS variants; 2PT: genotype composed by two PT variants

In Fig. 3, curves of survival (A) and retention of autonomy (B) are reported for all cases combined. The univariable Cox regression models are shown in the Additional file 2: Table S3 and Additional file 1: Fig. S3. In the final multivariable Cox regression model, the following variables were associated with a higher probability of death: *NHLRC1* PT/PT versus *EPM2A* all genotypes [HR 2.88; 95% CI 1.23–6.78] and Asian versus European origin [HR 2.63; 95% CI 1.07–6.49]. Late age at onset (18 + vs. < 18 years) was associated with a lower probability of death [HR 0.26; 95% CI 0.07–0.96]. Similar results, but with less power due to the smaller sample size, were observed for loss of autonomy (Table 3).

**Table 3:** Association of variables with death and loss of autonomy in *EPM2A-* and *NHLRC1-*related LD combined: multivariable Cox regression analysis

Variables	Death (n = 250)		Loss of autonomy (n = 116)	
	HR (95% CI)	P value	HR (95% CI)	P value
*NHLRC1*: MS/MS and MS/PT	Reference	–	Reference	–
*NHLRC1*: PT/PT	**2.88 (1.23**–**6.78)**	**0.015**	2.03 (0.75–5.56)	0.17
*EPM2A:* MS/MS and MS/PT and PT/PT	1.51 (1.10–6.39)	0.34	0.60 (0.18–2.07)	0.42
Age at onset 18 + versus < 18 years	**0.26 (0.07**–**0.96)**	**0.043**	0.25 (0.06–1.05)	0.058
Geographic origin				
Asian versus European	**2.63 (1.07**–**6.49)**	**0.036**	**2.80 (1.06**–**7.42)**	**0.038**
African versus European	4.30 (0.66–28.0)	0.13	7.18 (0.28–183)	0.23
American versus European	2.18 (0.73–6.5)	0.16	1.57 (0.61–4.02)	0.35

The statistically significant values (*p* < 0.05) are displayed in bold

## Discussion

In the present systematic review with individual participant data meta-analysis, we analyzed a large group of LD patients, demonstrating the existence of prognostic genetic factors in LD, namely the genotype defined according to the functional impact of the pathogenic variant. The *NHLRC1* genotype PT/PT was found to be significantly associated with a worse prognosis in terms of survival and loss of autonomy. On this basis, we could speculate that MS and PT variants in *NHLRC1* have a different impact on protein’s function and disease severity. Missense variants may lead to the production of a protein with some retained function, whereas truncating variants to a complete loss of function, as *NHLRC1* consists of only one exon. On the other hand, MS and PT variants in *EPM2A* do not appear to have this dychotomized behavior, possibly because truncating variants could still produce a low molecular weight protein with some retained functions, similarly to missense variants.

In addition, it could be argued that malin has a more multifaceted role and, as a result, its depletion leads to the most deleterious effect on brain glycogen metabolism [15]. The key function of laforin is to remove phosphate from glycogen and to date there have been no protein targets of laforin’s phosphatase activity [16]. Conversely, malin interacts with and/or ubiquitinates a number of proteins [16–23]. Most of the reported proteins are involved in either glycogen metabolism or endoplasmic reticulum stress response. Ubiquitination is one of the most common post-translational modifications of proteins and causes either degradation or change in function of the ubiquitinated protein depending on the type of ubiquitin chain linkage added to the protein. Malin can ubiquitinate proteins via a number of different linkage types, thus amplifying the complexity regarding the function of malin. For example, under certain circumstances malin ubiquitinates laforin via one type of ubiquitin linkage and triggers the proteosome degradation of laforin. Alternatively, the ubiquitination of glycogen phosphorylase by malin triggers nuclear accumulation of glycogen phosphorylase instead of trigging its degradation [15]. There are likely additional unknown proteins that malin ubiquitinates. Interestingly, *NHLRC1* has been recently found to be expressed at higher levels in the brain than in the major glycogen metabolizing organs, namely skeletal muscle, heart and liver, while the opposite emerged for *EPM2A* [24]. Thus, cellular data suggests that malin may have more multifaceted biological functions including on glycogen metabolism, endoplasmic reticulum stress, and cellular function. This is also consistent with recent data suggesting that laforin requires the presence of malin to fulfill some of its functions, and additional studies are required to determine the balance of dependent and independent roles of both malin and laforin [2]. Interestingly, the sub-analysis on the population carrying the missense homozygous p.Asp146Asn variant, confirmed a more favourable prognostic implication, with a disease duration greater than 15 years in all the patients. The slower disease course in patients with this genotype might be explained by a partial preservation of enzymatic activity of the p.Asp146Asn variant, since aspartic acid and asparagine share highly similar properties [6, 7].

The results of this study highlight the need for further research aimed at elucidating the intricate interplay between laforin and malin in the pathogenesis of LD. If a primary pathogenic factor can be confirmed, it could serve as a significant therapeutic target for the development of more effective drugs, in addition to inform genetic counseling. Furthermore, the finding that the *NHLRC1* genotype PT/PT is associated with faster disease progression should be taken into account when launching trials of disease-modifying therapies (e.g., by stratifying the population by genotype), to ensure that outcomes are correctly interpreted.

### Limitations

This review gives insight into the prognostic impact of genetic variants in the two LD-associated genes, however, it is possible that variants in other “modifier” genes might influence disease progression. The lack of this information in the examined papers may therefore limit the fields understanding of phenotypic variation.

As with other severe epilepsy syndromes, the optimization of the antiseizure medication therapy may allow temporary control of seizures and myoclonus and prevent complications such as status epilepticus. However, antiseizure medications do not influence the neurodegenerative process and are used in highly variable combinations between different patients and also in the same patient during disease course, thus were not considered in this prognostic review.

Death was reported in only one-fourth of included patients (69/250) and the specific cause only in a minority of them, thus not allowing any additional insight into the effect of genotype; this aspect might be taken into account in the setup of a subsequent study.

## Conclusions

This study demonstrates the existence of prognostic genetic factors in LD, namely the genotype defined according to the functional impact of the pathogenic variants. Although the reasons why *NHLRC1* genotype PT/PT is associated with a poorer prognosis have yet to be fully elucidated, it may be speculated that malin plays a pivotal role in LD pathogenesis.

### Supplementary Information

**Additional file 1. Text**: List of references included in the meta-analysis and search strategy. **Fig. S1**: Flow diagram representing the selection process of the included cases. **Fig. S2**: Subgroup analysis: homozygous p.Asp146Asn variant in *NHLRC1*. Survival in subjects homozygous for the p.Asp146Asn variant in *NHLRC1* according to Kaplan–Meier analysis. Legend: MS, missense pathogenic variants and small in-frame insertions/deletions; PT, nonsense, frameshift, splice site variants, and partial or total gene deletions. MS + PT: genotype composed by one MS variant and one PT variant; 2MS: genotype composed by two MS variants; 2PT: genotype composed by two PT variants. **Fig. S3**: Forest plot—Association of variables with death and loss of autonomy in EPM2A- and NHLRC1-related LD combined: univariable and multivariable Cox regression analysis (HR = Hazard Ratio). **Table S3**: Association of variables with death and loss of autonomy in EPM2A- and NHLRC1-related LD combined: univariable Cox regression analysis.

**Additional file 2. Table S1 **: Pathogenic variants in *EPM2A* [NM_005670.4] reported in the collected cases. Legend: (§) the standard HGVS nomenclature guidelines (https://varnomen.hgvs.org) were used to standardize the nomenclature of each variant, when possible. **Table S2**: Pathogenic variants in *NHLRC1* [NM_198586.3] reported in the collected cases. Legend: (§) the standard HGVS nomenclature guidelines (https://varnomen.hgvs.org) were used to standardize the nomenclature of each variant, when possible.

## Data Availability

Data supporting the findings of this study are available within the paper and its Supplementary Information.
